# Preschoolers’ Early Sentence Comprehension: Comparing Bilingual and Monolingual Children and the Role of Executive Function and Vocabulary Development

**DOI:** 10.1111/desc.70232

**Published:** 2026-06-11

**Authors:** Noorin Rodenhurst, Katherine Messenger

**Affiliations:** ^1^ Department of Psychology University of Warwick Coventry West Midlands UK; ^2^ Department of Psychology Lancaster University Lancaster Lancashire UK

**Keywords:** bilingualism, executive function, novel verbs, sentence comprehension, vocabulary

## Abstract

Previous research into children's comprehension of syntactic structures has investigated early awareness of transitive and intransitive structures amongst monolingual children but research into bilingual children's understanding of the same sentence structures is lacking. This study compared 46 3–5‐year‐old bilingual children who spoke English and an additional language with 64 monolingual, English‐speaking children, in their ability to associate transitive sentences with a causal scene, and intransitive sentences with a non‐causal scene. In contrast to previous studies, the current study used still images instead of videos to create eight trials per structure, increasing the number of trials and testing structure as a within‐participants manipulation. Children's vocabulary and executive function skills were also tested to observe interactions with the sentence comprehension task. Both groups showed the same overall pattern of effects, though the monolingual children were reliably more likely to match the causal scene to a transitive than bilingual children: Older children associated the transitive trials with causal scenes more than the intransitive trials, whereas 3‐year‐olds tended to associate both the transitive and intransitive sentences with causal scenes. Executive function performance did not differ between bilingual and monolingual children and was not related to sentence interpretation, but monolingual children had higher English vocabulary scores and children with greater vocabularies were better able to distinguish causal scenes as a match for transitive sentences. The results suggest that differences in comprehension of syntactic structures are more likely to be related to age and language ability, rather than differences in the number of languages spoken.

**Summary:**

- Bilingual and monolingual children showed similar patterns in their ability to comprehend transitive and intransitive sentences, though monolinguals more reliably associated transitives with causal scenes.
- 4‐ and 5‐year‐olds were better at associating transitives with causal scenes and intransitives with either causal or non‐causal, whereas 3‐year‐olds associated both sentences with causal scenes.
- Age was a better predictor of differences in interpreting transitivity than the number of languages children speak.
- No executive function differences between bilinguals and monolinguals were found, monolinguals had larger English verb vocabularies; vocabulary was related positively to causal scene/transitive associations.

## 1 Introduction

Bilingualism is seen as beneficial for children, not only for connecting them to both their heritage and community, but also for cognitive benefits, such as enhancing executive function skills (Bialystok 2011, 2018; Blom et al. 2014; Marian and Shook 2012). Nonetheless, there is still the perception amongst some parents of bilingual children that a language delay occurs (Abutbul‐Oz and Armon‐Lotem 2022; King and Fogle 2006; Lee et al. 2015), where a bilingual child's ability to understand and use a language may not reach the same level as a monolingual child of the same age (Dale et al. 2003; McLaughlin 2011; Whitehurst and Fischel 1994). One reason for this belief is that linguistic input is split between two languages, whereas monolingual children receive input in only one language (Hoff and Core 2013; Hoff and Ribot 2017; Hoff et al. 2014). The difference in input per language has been shown to be associated with slower growth in each individual language amongst bilingual children compared to their monolingual peers (Hoff et al. 2012). But at the same time, such differences do not necessarily indicate a delay in overall language development when both languages are considered together (Core et al. 2013). Recent evidence further indicates that bilingual and monolingual children may follow broadly similar early developmental trajectories, particularly in the timing of early language milestones, even where single‐language vocabulary growth differs (Muszyńska et al. 2026). Also, there is considerable variation in how much input bilingual children receive in each language, in which context linguistic input per language occurs, and from whom this input comes (Rowland 2013). Thus, there is likely variability in the degree to which bilingual language experiences influence language growth. Moreover, factors such as specific language and cognitive skills, for example, vocabulary development and executive function, which are related to language processing in important ways (Bialystok et al. 2010; Carlson and Meltzoff 2008; Hurtado et al. 2014; Messenger and Fisher 2018; Thothathiri et al. 2025; Woodard et al. 2016), may vary both within and across bilingual and monolingual populations. Here we consider how this variability in experience and development influence early sentence comprehension.

One way we can assess bilingual and monolingual children's early syntactic knowledge is to measure their understanding of sentences with novel verbs. Novel verbs do not provide semantic information to describe an event, therefore, to interpret these sentences, listeners must rely on grammatical knowledge to determine who is doing what to whom. As such, novel verb sentences test the availability of grammatical knowledge, that is, whether children have abstract knowledge of different sentence structures (Fisher et al. 2010; Yuan and Fisher 2009). In such studies, monolingual children can, for example, successfully identify a novel event that matches a novel verb sentence typically by 2–3 years of age (Chan et al. 2010; Dittmar et al. 2011; Dittmar et al. 2008a, 2014; Donnelly and Kidd 2021; Noble et al. 2011; Yuan and Fisher 2009). This suggests that monolingual children acquire verb‐general syntactic knowledge early on (Yuan and Fisher 2009). But little attention has been paid to whether bilingual children acquire such knowledge in the same way and at the same rate as monolingual children, despite the inevitable differences in their early linguistic input. Since input is crucial for driving the rate and manner of development, any circumstances in which that input may differ may lead to variation in development while differences in language and executive function skills may also relate to how sentence comprehension develops. This makes it critical to examine which aspects of development may be affected. This study is the first to explore bilingual and monolingual children's syntactic development by comparing their understanding of English novel verb sentence structures at different ages and by examining whether their sentence comprehension is related to their vocabulary and executive function abilities.

### 1.1 Children's Comprehension of Transitive and Intransitive Sentences

In acquiring the syntax of their first language(s), children must gather information about different types of verbs, the types of events they describe, and the syntactic structures used to express them. Different types of events, such as causal events, involving one participant acting on another, and non‐causal events, in which there is only one participant, are described by different types of structures. Transitive verbs (e.g. *chase*) describe a causal relationship between an agent and a patient thus causal events are described by transitive structures involving two nouns. Typologically, most languages in the world express this in sentences with a subject‐object‐verb structure or a subject‐verb‐object (SVO) structure, though all word order permutations are possible and attested (Greenberg 1963). In English, these are expressed in sentences with a SVO structure, wherein the subject appears first in the sentence and corresponds to the agent of the scene carrying out the action described by the verb, while the object appears post‐verbally and corresponds to the patient, having the action described by the verb done to them. In English, the meaning of such sentences can therefore be deduced via word order, using the first argument as agent word order cue (Abbot‐Smith and Serratrice 2015; Chan et al. 2009; Gibson et al. 2013; Rowland 2013).

Intransitive verbs (e.g., *sleep*) describe an event involving only an agent, that is, a non‐causal event, though multiple agents can be involved. Intransitive verbs can be both specific, such as *jump*, and general, such as *exercise*. Thus, non‐causal events are described by intransitive structures involving one noun phrase, which can be a conjoined noun phrase. In English, the verb‐final nature of this structure should provide speakers with sufficient syntactic information to distinguish them from transitive sentences, which are verb‐medial (Gertner and Fisher 2012). In other languages, case‐marking is a more reliable cue than word order for speakers to identify the agent and patient of transitive sentences, for example German (Brandt et al. 2016; Özge et al. 2022), and Hindi (de Hoop and Narasimhan 2005; Montrul et al. 2019; Verbeke and De Cuypere 2009). Meanwhile, in languages with flexible word order, making it an unreliable cue, speakers rely solely upon case‐marking for sentence comprehension in transitive and intransitive sentences, for example, Japanese (Kayama and Oshima‐Takane 2022; Okuno et al. 2020).

Across several studies, monolingual children have been found to successfully associate novel‐verb transitive sentences (e.g., *the duck is blicking the bunny*) with a causal event at 2 years (Chan et al. 2010; Dittmar et al. 2011; Dittmar et al. 2008a, 2014; Göksun et al. 2008; Naigles 1990; Naigles and Kako 1993; Noble et al. 2011), and as early as 22 (Messenger et al. 2015), 21 (Arunachalam et al. 2013; Gertner and Fisher 2012) or even 19 months (Yuan et al. 2012). Additionally, young monolingual children distinguish transitive sentences from intransitive sentences (e.g., *the duck is blicking*) for which they do not show a preference for a causal event. These studies used novel verbs, which provide no semantic information to inform children's understanding of the sentence. Children's ability to use the grammatical structure in order to interpret the sentence and match it to a referent scene, known as *syntactic bootstrapping*, implies early abstraction of grammatical knowledge (e.g., Fisher et al. 2010; Fisher et al. 2020). In the syntactic bootstrapping account, young children acquire early verb general knowledge, such as the argument structure and participant roles of different types of verbs, for example, transitive verbs, and use this to guide future verb learning experiences. Here, we exploit this paradigm as a means to test whether young bilingual and monolingual children have verb‐general knowledge for basic syntactic structures.

Results for intransitive sentences are more mixed: some studies have found that 3‐year‐olds associate these with a non‐causal event (Noble et al. 2011), but others find that children show no preference for a non‐causal event over a causal event, performing at chance (Arunachalam and Waxman 2010), even at 3 or 4 years of age (Arunachalam et al. 2016; Noble et al. 2016; see also Cao and Lewis 2022). These findings of at‐chance performance on intransitive sentences could be experimental artifacts for this type of sentence, rather than evidence of children lacking syntactic knowledge. Such sentences could entail a verb with a more general meaning that could apply to the whole of a causal scene, or alternatively they could entail a verb that describes aspects of an event even if it does not describe the entire event. For example, in a causal scene depicting a character being pushed, the novel‐verb intransitive could have a meaning such as ‘exercising’ or ‘playing’ describing what both characters are doing, or it could legitimately describe the movement of one or other character (e.g. bending, stretching) without describing the entire scene (Arunachalam and Waxman 2010; Yuan and Fisher 2009). Thus, a causal event could be as good a referent for an intransitive sentence as a non‐causal event, whereas a non‐causal event is never a good referent for a transitive sentence. Indeed, adults, whose verb‐general knowledge is not in doubt, have also shown chance performance on such trials (Arunachalam et al. 2016). Critically, children's performance with transitive and intransitive sentences tends to differ reliably, suggesting that they do distinguish these syntactic forms, even if matching the latter sentence to a referent scene is more ambiguous in these tasks.

One type of novel‐verb intransitive test sentence may present particular difficulty for young children: some studies use a conjoined agent (e.g. *the duck and the bunny are blicking*) to control the number of characters across sentence conditions. For children still developing the appropriate syntactic representations for transitive and intransitive sentences, this could create an ambiguity as to whether the sentence refers to a causal or non‐causal event because the sentence contains two nouns. Gertner and Fisher (2012) found that 21‐month‐old monolingual children looked more at a causal event than a non‐causal event when hearing a conjoined‐agent intransitive sentence where the first named agent was the agent of the causal event, and they looked at the causal event as often as they did for transitive sentences. When the first agent in the intransitive sentence was the patient in the causal event, participants looked at the causal event less often than participants in the transitive and agent‐first intransitive conditions. This suggests that children interpreted those agent‐first intransitive sentences as causal, assigning agent/patient relationships according to the word order of the two nouns (but see Jiang and Haryu 2014, for different results in Chinese).

Note that, while many of the above‐cited findings were from studies testing monolingual English‐speaking toddlers, early verb‐general knowledge has been observed consistently across children from a range of monolingual language backgrounds. For example, German‐speaking 21‐month‐olds looked longer at a causal event than a non‐causal event when hearing a novel‐verb transitive, though only when the warm‐up phase included transitive sentences with known verbs (Dittmar et al. 2008b). Similarly, in a forced‐choice pointing task, Jiang and Haryu (2014) found that the monolingual Chinese‐speaking 2–4‐year‐olds could map transitive sentences to causal events but even the oldest children did not associate the non‐causal event with intransitive sentences (see also Lee and Naigles 2008). Parallel findings have been observed in other argument dropping languages, such as Turkish (Göksun et al. 2008) and Japanese (Matsuo et al. 2012). Arunachalam et al. (2016) compared monolingual children and adults’ comprehension of intransitive sentences in English and Mandarin. Both groups performed at chance with bare intransitive sentences but did associate an intransitive sentence that had a semantic modifier meaning *together*/*each* with a non‐causal event, supporting the idea that inferring the intended referent of the intransitive is difficult without further cues. Some studies do highlight crosslinguistic differences in *when* syntactic abilities emerge. For example, Jiang and Haryu (2014) found that even at 5 years, Chinese‐speaking children did not associate intransitive sentences with non‐causal events (whereas some 3‐year‐olds do so in English, Noble et al. 2011). They suggested that argument dropping may delay understanding of intransitive sentences—whilst a Chinese sentence with two arguments can only be transitive, a Chinese sentence with one argument may be intransitive, or transitive with the argument dropped. Unlike monolingual English‐speaking toddlers, 2‐year‐olds acquiring Korean did not show learning from novel verb transitive sentences with and without overt subjects suggesting they were unable to comprehend these sentences correctly (Arunachalam et al. 2013), however by 4–5 years of age, Korean‐learning monolingual children did pass such tests (Shi et al. 2023). Crosslinguistic reliability of the cues that signal argument structure may explain these age‐related differences (see also Chan et al. 2009).

The above evidence is focused on children acquiring language in monolingual environments. The path of development for children acquiring two or more languages remains to be investigated. This is an important oversight in understanding children's language development, since 40%–70% (estimates vary) of the world's population are bilingual (Byers‐Heinlein et al. 2019; Grosjean 2021, 2024). Bilingual children who are acquiring two languages must learn how each language structures transitive and intransitive sentences, but they experience comparatively less input than monolingual children from which to do so. Therefore, an important question to address is: do bilingual children develop verb‐general knowledge of basic syntactic structures at the same rate that monolingual children do?

### 1.2 The Role of Vocabulary Knowledge and Executive Function in Sentence Comprehension

Sentence comprehension abilities are influenced by and related to other language skills and cognitive abilities (Kidd and Donnelly 2020). For example, lexical and syntactic processing are positively correlated with vocabulary in monolingual (Borovsky et al. 2012; Fernald et al. 2006; Messenger and Fisher 2018) and bilingual children (Hurtado et al. 2014). Studies that examine early sentence comprehension, such as those reviewed above, often observe an association of vocabulary with sentence comprehension (e.g., Dittmar et al. 2011; Donnelly and Kidd 2021; Huang et al. 2017; Messenger and Fisher 2018). This may be because a larger vocabulary size indicates a more advanced stage of language development, by which children are more likely to have acquired the relevant structure, or more efficient sentence processing abilities, and therefore are more able to predict or integrate the appropriate syntax (Hurtado et al. 2014; Messenger and Fisher 2018).

A substantial body of research highlights the strong relationship between vocabulary growth and grammatical development in early bilingual language acquisition. Studies have shown that vocabulary size is a robust predictor of emerging grammatical abilities, with evidence suggesting that lexical and morphosyntactic development are closely linked in early childhood. For example, Marchman and Martínez‐Sussmann (2004) demonstrated that in bilingual children, growth in productive vocabulary is strongly associated with the development of grammatical structures in each language. Similarly, Hoff et al. (2018) found that differences in bilingual children's knowledge, particularly related to differences in each language's exposure, can predict corresponding differences in grammatical development. These findings suggest that children's vocabulary size is not just an indicator of general language proficiency but may play a role in supporting developing sentence comprehension. This leads us to expect that children with larger vocabularies will show more robust mappings between syntactic structure and events, regardless of language background.

Another factor relevant to sentence comprehension skills is executive function. In monolingual speakers, development of executive function is thought to be related to improved sentence revision (Thothathiri et al. 2025; Woodard et al. 2016) and reduced perseveration in processing (Mazuka et al. 2009). Many argue that bilingual children have an advantage in executive function skills, where the bilingual experience naturally enhances cognitive abilities (Bialystok and Viswanathan 2009). Three elements of this cognitive skill are relevant to bilingual language: *monitoring*, being aware of the language currently in use; *switching*, being able to successfully switch from one language to another; and *inhibition*, being able to ignore the set of rules of one language and attend to the other (Bialystok and Viswanathan 2009; Miyake et al. 2000). Much research supports the idea of an executive function advantage in bilinguals over monolinguals (Bialystok 2011, 2015; Bialystok and Martin 2004; Carlson and Meltzoff 2008; Foy and Mann 2014; Grote et al. 2021; Zelazo 2006), though others have found no difference between bilingual and monolingual children (Arizmendi et al. 2018; Cespón and Carreiras 2020; De Cat et al. 2018; Lowe et al. 2021; Paap et al. 2015; Ross and Melinger 2017). Despite the disagreement in the literature, the possibility remains that enhanced executive function skills, due to learning and using two sets of syntactic rules in sentence comprehension, would support bilingual children to attend to syntactic rules of the language in use and ignore the language not in use, thus children who have better executive function skills may perform better in a sentence comprehension task.

### 1.3 The Present Study

We compared monolingual English‐speaking children and bilingual children who speak English and any other language, in their comprehension of transitive and intransitive sentences with novel verbs in English; to the best of our knowledge, this is the first study to do so. We compared 3, 4, and 5‐year‐olds to examine whether accuracy in comprehension improved with age. Previous research measuring children's interpretation of transitive and intransitive sentences has been typically conducted with children between 2 and 4 years of age (Chan et al. 2010; Dittmar et al. 2011; Dittmar et al. 2008a 2014; Donnelly and Kidd 2021; Noble et al. 2011; Yuan and Fisher 2009). We chose a slightly older age range as we anticipated that bilingual children may show more reliable effects at these ages, and both groups might show changes in their interpretation of conjoined intransitives between 3 and 5 years of age. We assessed children's English vocabulary using the verb comprehension measure of the British English Cross‐linguistic Lexical Tasks (CLTs) (Haman et al. 2012) and executive function with the (nonverbal) Bivalent Shape Task (BST) (Mueller and Esposito 2014) to examine how these factors related to sentence comprehension.

This study also extends previous research by comparing children's comprehension of two structures within‐participants over a greater number of test trials. Studies assessing syntactic knowledge often include several trials to familiarise young children with the nature of the experiment; therefore, to avoid fatigue in young participants, only a small number of test trials are typically included. There could be as few as one (Yuan and Fisher 2009), two (Chan et al. 2010; Dittmar et al. 2008a, 2014; Donnelly and Kidd 2021; Noble et al. 2011), three (Dittmar et al. 2011), or four (Arunachalam et al. 2016; Gertner and Fisher 2012; Messenger et al. 2015; Noble et al. 2016) test trials, although Jiang and Haryu (2014) and Arunachalam et al. (2013) used six and Abbot‐Smith and Serratrice (2015) used nine. With a small number of trials, any issues with the stimuli may not be easily observed and reduced performance that is assumed to be associated with the sentence structure may in reality be related to the item (Ambridge and Rowland 2013); it is difficult to claim that participants performed at chance when, for example, they answered correctly on one out of only two trials. A greater number of trials may help to avoid item‐effects and give younger children more opportunity to better demonstrate their abilities. Furthermore, all the above studies tested structures between participants (cf. Gertner and Fisher 2012; Noble et al. 2011), such that participants were tested on either transitive or intransitive sentences. Within‐participant manipulations are more powerful and allow us to compare the same individuals’ knowledge of each form, controlling for differences in development. To address these issues, we adapted the video stimuli from Noble et al.’s (2011) study, using static images of scenes captured from the videos to create a greater number of transitive and intransitive trials. Though most studies of this nature use video stimuli, Abbot‐Smith and Serratrice (2015) demonstrated that still images make a suitable alternative, particularly in studies with a greater number of trials.

As in other preferential‐pointing syntactic bootstrapping studies, we presented children with a novel verb sentence (e.g., *the duck is blicking the bunny*) and two candidate referents—two images of novel events, one of which depicted a casual event involving two participants, (e.g. an agent (the duck) acting on a patient (the bunny)) and one of which depicted a non‐causal, simultaneous action event involving two participants (the duck and the bunny) both as agents carrying out the same movement. The correct response when listening to a transitive sentence is to point to the causal event. When listening to an intransitive sentence, participants could be more likely to point to the non‐causal, simultaneous action event, however, as observed in previous research, participants may also point to the causal event if interpreting the intransitive sentence as having a more general meaning. Thus, following intransitive sentences, participants’ referent selection could be at chance but following transitive sentences it should be reliably above chance and towards the causal event.

Based on monolingual evidence, we expected that children would point at a causal event more often when hearing a transitive sentence. Based on previous research we predicted that when hearing an intransitive sentence, their points could be at chance, at least in the youngest age groups; alternatively, they may interpret the conjoined agent as two participant roles and point more at the causal event. We predicted that based on their generally reduced input, bilingual children would be less accurate in matching these structures to events than monolingual children.

This study also examined bilingual and monolingual children's English vocabulary size: we predicted that monolingual children would have higher English vocabulary scores than bilingual children (Bialystok 2010), since the vocabulary measure only captured a subset, the English part, of the bilingual children's vocabularies. We also expected vocabulary scores to be related to performance on the sentence comprehension task: we predicted that those with greater vocabulary would perform more accurately on the sentence comprehension task (e.g. Dittmar et al. 2011; Donnelly and Kidd 2021; Hoff et al. 2018; Huang et al. 2017; Marchman and Martínez‐Sussmann 2004; Messenger and Fisher 2018; Paradis 2011; Paradis et al. 2017). Finally, we examined differences in executive function: we predicted that bilingual children would show an advantage over monolingual children (Bialystok 2011, 2015; Bialystok and Martin 2004; Carlson and Meltzoff 2008; Foy and Mann 2014; Grote et al. 2021; Zelazo 2006). We also assessed whether executive function is related to young children's ability to comprehend sentences. We predicted that those with superior inhibition and switching abilities might be more accurate in reaching the correct interpretation on the sentence comprehension task (Wolleb 2015; Woodard et al. 2016).

## 2 Methodology

### 2.1 Participants

#### 2.1.1 Bilingual Participants

Fifty‐two bilingual children who spoke English and one other language were recruited via social media posts, childrenhelpingscience.com (Scott and Schulz 2017; Sheskin et al. 2020), and the Warwick Research with Kids Group family database. The database consists of families who were recruited from events held in the urban/suburban cities of Birmingham and Coventry, UK, with most families living in those cities. Six children were excluded as they either did not pass the screening stage of the task (4, see below), or they did not complete all the tasks (2). This left a final group of 46 bilingual participants (mean age: 4;5; SD: 10.75 months; range: 3;0–5;11; 26 females) from different language backgrounds (see Table 1). Of this final group, 16 were 3‐year‐olds, 13 were 4‐year‐olds, and 17 were 5‐year‐olds. We aimed to test approximately 20 participants per age group, in line with Noble et al. (2011) and Arunachalam (2016) who conducted similar sentence comprehension tasks, but we were unable to quite reach this target for bilingual participants, which may limit our power to detect effects within each age band. Based on the highest level of maternal education, as answered in a parental questionnaire, 42 participants were from high socioeconomic status (SES) backgrounds, and four participants were from low SES backgrounds. Maternal education at bachelor's degree level and above were deemed high‐SES, below this was determined to be low‐SES. All participants but one were based in the UK, one was based in the US.

**TABLE 1:** The number of bilingual speakers for each language by order of acquisition.

Languages	First language	Second language
English	40	6
French	—	5
Spanish	3	4
German	—	4
Urdu	—	4
Welsh	—	4
Greek	—	3
Portuguese	—	3
Italian	1	2
Gujarati	—	2
Polish	—	2
Punjabi	—	2
Romanian	1	—
Ukrainian	1	—
Arabic	—	1
Bengali	—	1
Catalan	—	1
Kannada	—	1
Shona	—	1

#### 2.1.2 Monolingual Participants

Seventy‐two monolingual English‐speaking participants were also recruited via the same means. Eight were excluded as they did not pass the screening stage (7) or did not finish all the tasks (1). This left a final group of 64 (mean age: 4;6; SD: 11.74 months; range: 3;0‐5;11; 27 females). In this final group, there were 21 3‐year‐olds, 19 4‐year‐olds, and 24 5‐year‐olds. Fifty‐seven participants were categorised as high‐SES and seven participants as low‐SES. These participants were identified as monolingual in a pre‐study parental questionnaire: parents stated that English was their child's only known language.

### 2.2 Design

This study used a repeated measures design with two independent variables. Sentence type (2: transitive or intransitive) was manipulated within‐participants, and language group (2: bilingual or monolingual) was a between‐participants factor. The dependent variable for all tasks was the number of correct points to the target image on screen.

### 2.3 Materials

#### 2.3.1 Sentence Comprehension Task

This study used the forced choice pointing task created by Noble et al. (2011), but the task was adapted for an online context, and the number of transitive and intransitive trials was increased. The original sentence comprehension task contained two trials each for transitive and intransitive sentences (and a further four for trials testing semantic role assignment, not tested here). We increased this to eight per structure for the current study by using the eight novel verbs from the original task (*blick, glorp, meek, wug, klimp, dax, krad*, and *jemm*), and adding a further eight novel verbs: two of these, *filp* and *pilk*, were taken from Noble et al. (2016) and another novel verb, *moop*, was taken from Rowland and Noble (2010), while five novel verbs were newly created for this task: *rill*, *nav*, *pake*, *ginde*, and *jeed*. These new novel verbs were created by taking existing, single syllable words in English and changing one phoneme to create a word that does not exist (i.e., *pill* to *rill, nap* to *nav, poke* to *pake, find* to *ginde*, and *need* to *jeed*). Eight of the novel verbs were assigned to transitive items and eight were assigned to intransitive items.

Still images were taken from the video stimuli created by Noble et al. (2011). Two screenshots per four videos were taken and these image pairs were also flipped horizontally to create a total of 16 items. Transitive stimuli showed two characters participating in a causal event, for example, a duck pushing a rabbit's head downwards. These events were accompanied by audio giving the transitive sentence with full noun phrases, for example, “*Look, the duck is klimping the rabbit*”; sentences were recorded by the first author who is a native English speaker with a standard British English accent. Intransitive trial stimuli showed two characters participating simultaneously in the same non‐causal event, for example, a duck and a rabbit lifting their arms. The audio gave the intransitive sentences with conjoined full noun phrases, for example, “*Look, the duck and the rabbit are ginding*”; see Appendix for a full item list.

**FIGURE 1:**
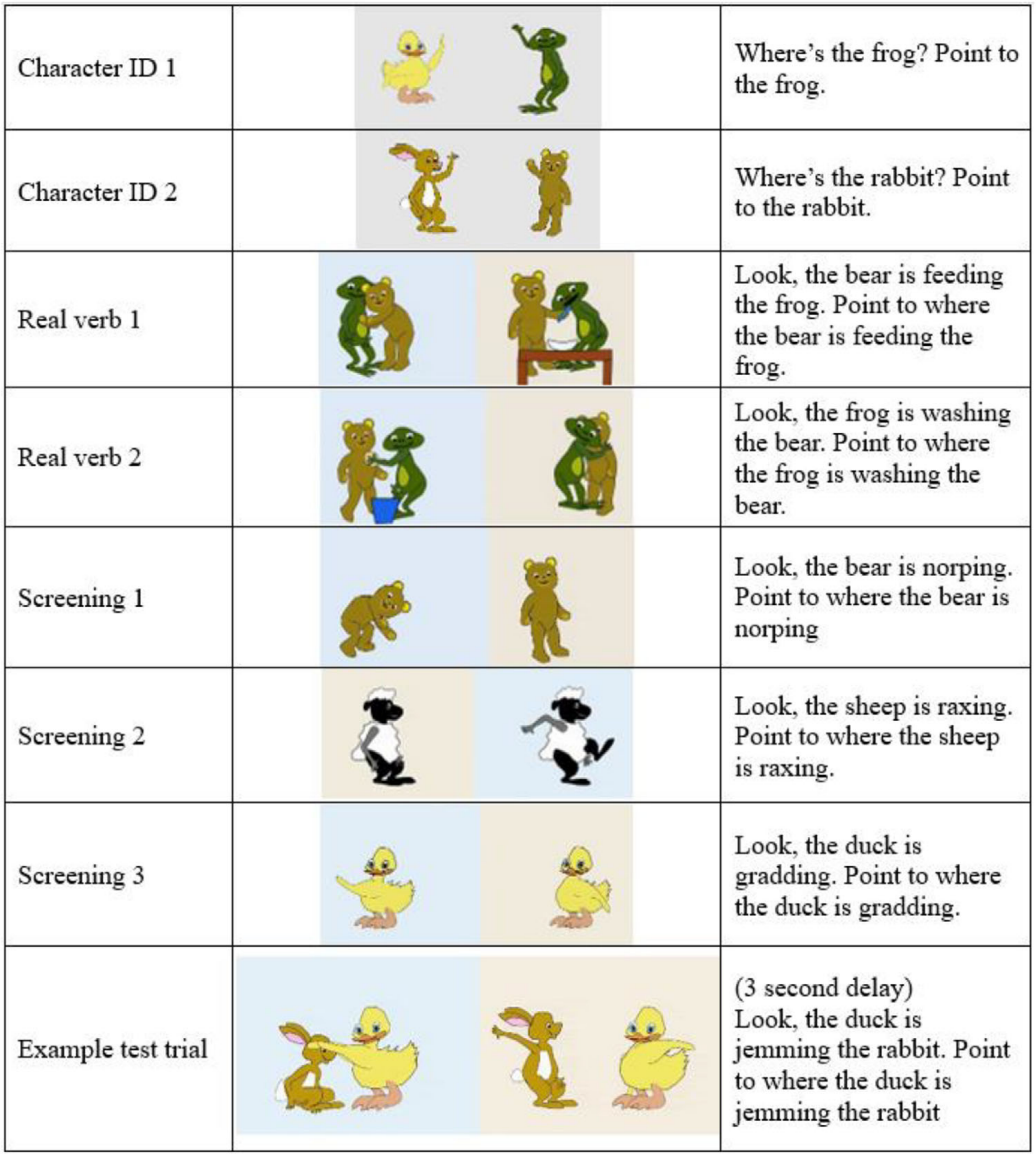
Example images and sentences for each trial type.

On each test trial, a causal event was paired with a non‐causal event with the same characters, which appeared side‐by‐side on the screen for 16 seconds (see Figure 1). First participants completed two intransitive target trials, followed by two transitive target trials, with subsequent trials alternating between the two structure pairs, until eight trials per structure had been presented to participants. We counterbalanced which side the target appeared across trials.

Before the test items, participants completed a set of practice and screening trials. First, participants saw two character identification trials (4 s) in which two animals were presented and were instructed to point to the target character (Figure 1).^1^ Participants then saw two trials (10 s) with real transitive verbs in which participants viewed two causal events, for example, a bear tickling a frog and a bear feeding a frog, and heard audio matching one of them. Participants then completed three screening trials (9 s) in which they saw a single character on both sides of the screen; on one side the character stood passively and on the other, the character performed an action, such as lifting their arms. Participants heard a novel‐verb intransitive describing the scene where an action was taking place. Immediately after these trials the participants began the test trials.

Audio‐recordings played automatically at the start of each trial, repeating once if 10 s passed with no response. Children were instructed to point to the image they believed matched the sentence they heard, and parents were asked to record their response by pressing the left arrow key if their child pointed to the left image, or the right arrow key if their child pointed to the image on the right. Each key press was automatically recorded on Pavlovia. Parents were reminded not to help their child or tell their child the correct answer.

#### 2.3.2 Cross‐linguistic Lexical Tasks

As this study measures verb‐general knowledge, we used the verb comprehension subtest of the British English CLT (Haman et al. 2012). CLTs are designed to be suitable for use with both monolingual and bilingual children due to each language measure being developed using the same procedure and criteria and using culturally‐appropriate stimuli, avoiding the issues with translating measures (Haman et al. 2015). Data from monolingual children suggest that the CLTs are an appropriate measure for assessing vocabulary knowledge in children younger than 6 (Haman et al. 2017). The English CLT has been found to correlate well with other measures of language proficiency (Komeili et al. 2023) as well as parental assessment (Hansen et al. 2019) for bilingual children. For each trial, four separate events were displayed on the screen, and one was described with a question, for example, ‘*Who is running?*’, and the child chose which scene they thought matched the audio. All test sentences were recorded by the experimenter; audio played automatically at the start of each trial and was repeated once if no response was made after 10 s. The scenes were numbered from 1 to 4, and parents were asked to press the number key that matched their child's pointing response.

#### 2.3.3 Bivalent Shape Task

The BST (Mueller and Esposito 2014) was used to measure executive function. This task is entirely non‐linguistic, making it suitable for testing the Stroop effect amongst younger children, as well as for comparing across participants with different language backgrounds and allowing those with weaker English language comprehension to carry out the task. The BST shows good reliability across items within the task (Buhrs et al. 2024). Participants viewed a large circle or square in the middle of the screen, a red circle towards the bottom left of the screen, and a blue square towards the bottom right of the screen. Children must select the small image which matches the shape of the large image on each trial. Across conditions, the colour congruity between shapes is manipulated: on neutral trials the large shape has no colour; on congruent trials, the large circle was red, and the large square was blue, matching the colour of the small shapes; on incongruent trials, the large circle was blue, and the large square was red, creating a colour‐shape mismatch, that is, a Stroop effect (Stroop 1935), with the small shapes. On incongruent trials, participants must inhibit responding by colour and only respond by shape.

Children received visual feedback on each trial (note that in the original task, the feedback was auditory): the correct shape was circled on screen, regardless of whether the child's answer was correct. There was no time limit for the trials to avoid recording no response trials. Children were instructed to point to the small item they thought matched the large item and parents were asked to record the answer by pressing the Z key if the child chose the shape on the left and the M key if they chose the shape on the right.

### 2.4 Procedure

All the above tasks were created in PsychoPy, version 2020.2.6 (Peirce et al. 2019) and hosted on Pavlovia (pavlovia.org). The study took place online, rather than in person, due to the COVID‐19 pandemic. Parents were able to carry out the study with their child at their own convenience using a laptop or computer. Parents were emailed a link to a Qualtrics survey, which included an information sheet, consent form, and short questionnaire asking demographic information. This included information on languages spoken by the child, parents’ highest level of education, the country they currently resided in and whether the child and the parents had been born in that country, or how long they had lived in their current country of residence.

Parents were then emailed a unique Pavlovia link for them to complete the study with their child. Participants were assessed in a single testing session at a time and place of their parents’ choosing. Parents were advised to choose a quiet environment for testing to take place. Parents were instructed before the tasks and in between each task not to correct their child's mistakes during the tasks and that it is acceptable for their child to answer incorrectly; once a response was chosen on each task, it could not be changed. The order of tasks was sentence comprehension, BST, and CLT verb comprehension for each child. Once all tasks had been completed, this was recorded in Pavlovia, and parents were then emailed with debriefing information, a certificate, and activity pack for their child.

## 3 Results and Analysis

For the character identification, real verb and screening trials, responses were coded as 1 if children pointed to the correct, matching image, and 0 if they pointed to the non‐matching image. For the test items, responses were coded as 1 if children pointed to the causal event and 0 if they pointed to the non‐causal event. For the vocabulary and executive function tasks, correct responses were coded as 1 and incorrect responses as 0. Age in months was included as a continuous variable in the analysis to look for developmental trends, but for presentation purposes, the results have been grouped by age in years. First, we analysed the sentence comprehension task results alone, then we added children's vocabulary and executive function scores into the analyses to investigate whether their sentence comprehension results were related to these factors.

### 3.1 Bilingual and Monolingual Sentence Comprehension

Both bilingual and monolingual participants of all ages almost reached ceiling on the character identification, real verb, and screening trials (see Table 2). This suggests that participants understood the nature of the task before they carried out the test trials; participants who failed to reach at least two out of three correct responses for the screening trials were removed from analysis, as per Noble et al. (2011). Table 2 also shows that on transitive and intransitive test trials, participants in both groups typically pointed more frequently towards the causal event for transitive than intransitive sentences.

**TABLE 2:** Mean number (SD) of points by participant group and age to the target image for character identification (2 trials), real verb (2 trials), and screening (3 trials), and mean proportion points to the causal event for transitive (8 trials) and intransitive (8 trials) sentences.

Participants	Character ID	Real verbs	Screening	Transitive	Intransitive
Bilingual (all)	1.98 (0.15)	1.83 (0.44)	2.63 (0.49)	0.72 (0.21)	0.56 (0.21)
3‐year‐olds	2.00 (0.00)	1.81 (0.40)	2.62 (0.50)	0.69 (0.20)	0.63 (0.21)
4‐year‐olds	2.00 (0.00)	1.85 (0.38)	2.62 (0.50)	0.72 (0.16)	0.51 (0.21)
5‐year‐olds	1.93 (0.27)	1.82 (0.53)	2.65 (0.49)	0.76 (0.25)	0.54 (0.22)
Monolingual (all)	1.98 (0.13)	1.86 (0.35)	2.59 (0.50)	0.84 (0.17)	0.56 (0.25)
3‐year‐olds	2.00 (0.00)	1.81 (0.40)	2.71 (0.43)	0.80 (0.14)	0.64 (0.19)
4‐year‐olds	2.00 (0.00)	1.74 (0.45)	2.47 (0.51)	0.85 (0.19)	0.52 (0.22)
5‐year‐olds	1.96 (0.20)	2.00 (0.00)	2.58 (0.50)	0.86 (0.18)	0.52 (0.31)

The responses were analysed in logistic mixed effects (LME) models to predict participant points to the causal event with sentence type, language group, and age in months (36–71 months) as fixed effects and all interactions. Sentence type (intransitive = −0.5, transitive = 0.5) and language group (bilingual = −0.5, monolingual = 0.5) were sum coded and centred to have a mean of 0 and a range of 1; age in months was converted to Z scores. The models included by participant random slopes for sentence type, and participant and item random effects. Analyses were carried out in R (R Core Team 2022) using the lme4 package (Bates et al. 2015); we used the report package to obtain 95% confidence intervals for all fixed effects (Makowski et al. 2023). We fit maximal models (Barr et al. 2013), simplifying the random effects structure until a model converged; see Table 3 for the output from the analyses. All data and analysis code are available on OSF: https://osf.io/gdvcu/?view_only=82d1e2aad30c488ea767303da6597e4f.

**TABLE 3:** Logistic mixed effects models for bilingual and monolingual sentence comprehension.

Predictor	*β*	SE	95% CI	*Z*	*p*
**Model 1 – Bilingual and monolingual sentence comprehension** ^a^					
Intercept	0.89	0.12	[0.66, 1.13]	7.45	<0.001
Sentence type	1.23	0.24	[0.75, 1.71]	5.03	<0.001
Language group	0.38	0.15	[0.08, 0.68]	2.45	0.014
Age in months	−0.01	0.08	[−0.01, 0.01]	−0.10	0.920
Sentence type × language group	0.80	0.32	[0.17, 1.43]	2.49	0.013
Sentence type × age in months	0.48	0.16	[0.17, 0.79]	3.01	0.003
Language group × age in months	−0.11	0.15	[−0.41, 0.19]	−0.71	0.477
Sentence type × language group × age in months	0.11	0.32	[−0.51, 0.73]	0.35	0.727

Model 2 – Bilingual sentence comprehension^b^					
Intercept	0.68	0.11	[0.46, 0.91]	5.95	<0.001
Sentence type	0.82	0.23	[0.38, 1.27]	3.62	<0.001
Age in months	0.00	0.01	[−0.01, 0.02]	0.46	0.643
Sentence type × age in months	0.04	0.02	[0.00, 0.07]	1.99	0.047

Model 3 – Monolingual sentence comprehension^c^					
Intercept	1.11	0.16	[0.78, 1.43]	6.71	<0.001
Sentence type	1.65	0.34	[0.98, 2.32]	4.82	<0.001
Age in months	−0.01	0.01	[−0.03, 0.01]	−0.60	0.546
Sentence type × age in months	0.05	0.02	[0.01, 0.09]	2.30	0.021

^a^ Response∼Sentence type*Language group*age_months + (1+Trial|Ppt_ID) + (1|ItemNo).

^b^ Response∼Sentence type*Centerage_months + (1+Trial|Ppt_ID) + (1|ItemNo).

^c^ Response∼Sentence type*Centerage_months + (1+Trial|Ppt_ID) + (1|ItemNo).

**FIGURE 2:**
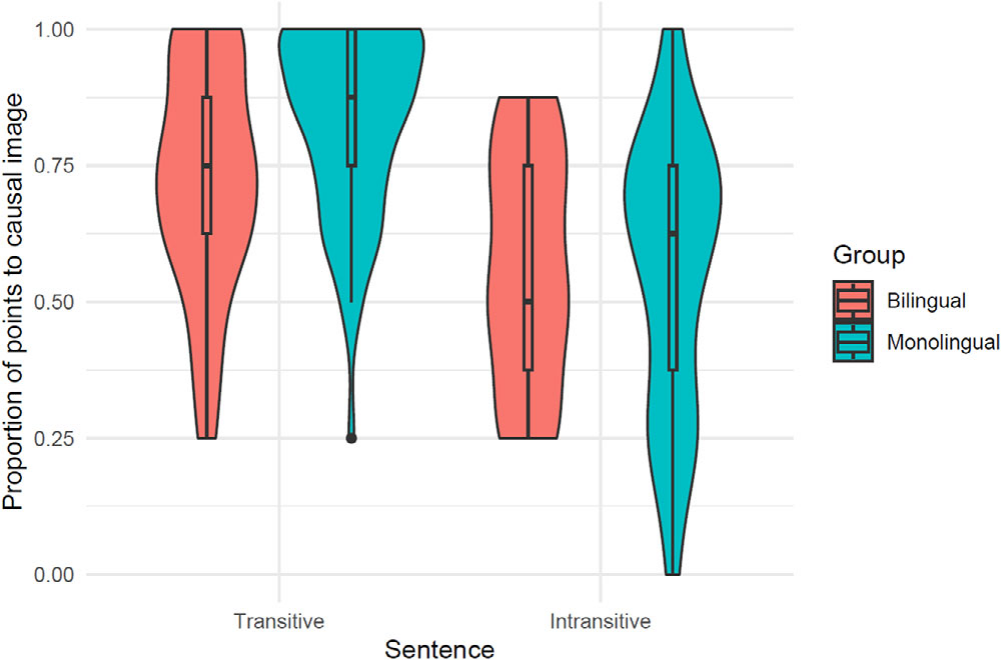
Proportion of points to the causal event in the transitive and intransitive sentence task by language group. The box represents the interquartile range, the thick line represents the median score, and the whiskers represent the minimum and maximum scores.

The converging model included participant and item random effects, and participant random slopes for sentence type. The main effect of sentence type was significant, (see Table 3, model 1), indicating that the children pointed more at the causal event for transitive sentences than for intransitive sentences (see Figure 2). There was a significant main effect of language group: the monolingual children pointed more at the causal event than the bilingual children, irrespective of sentence type. Age in months was not significant.

**FIGURE 3:**
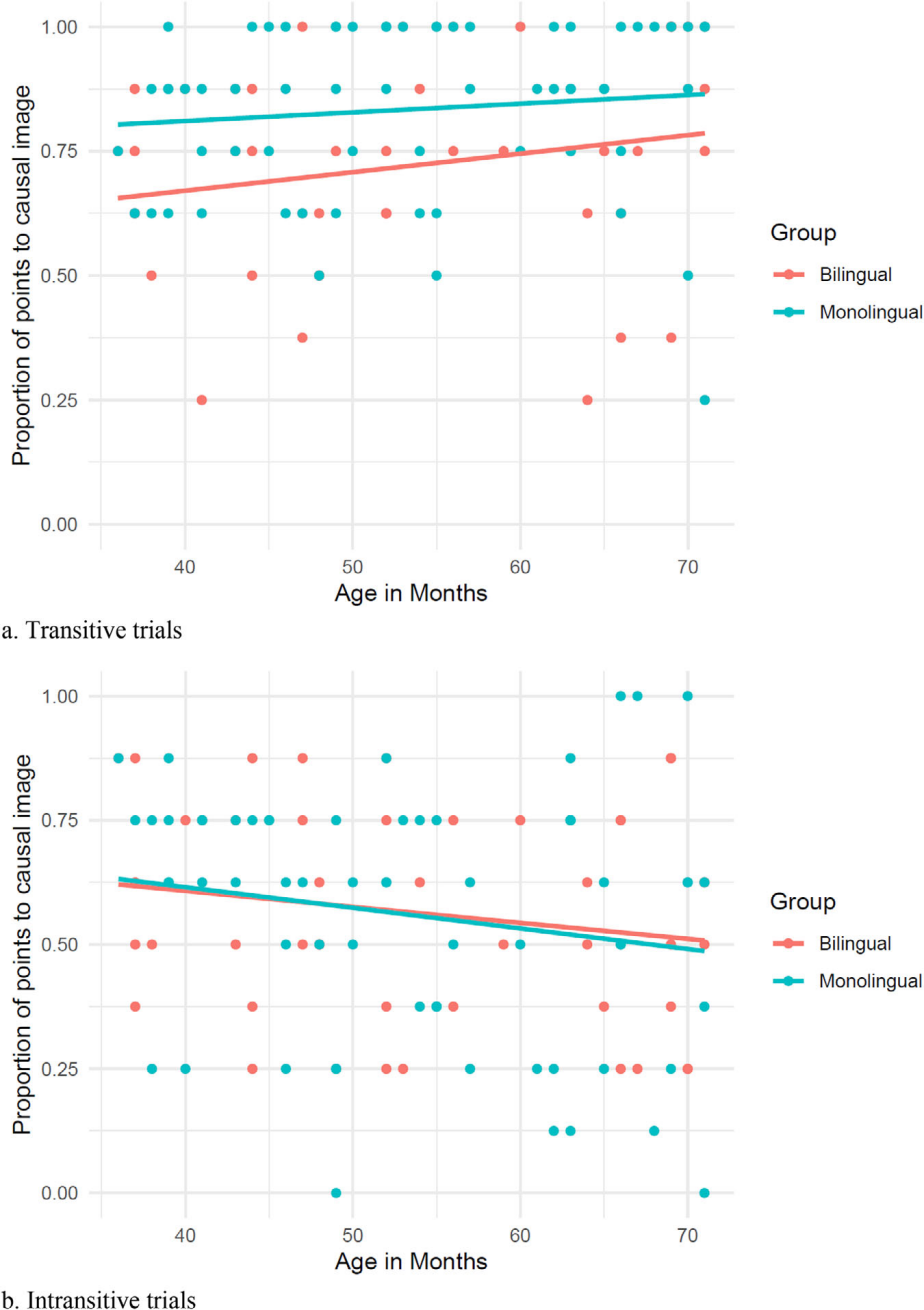
Relationship between age in months and points to the causal event in the transitive (a) and intransitive (b) trials for bilingual and monolingual children.

There was a significant interaction between sentence type and language group, with monolinguals more likely to point to the causal event during transitive than intransitive sentences, than bilinguals. The interaction between sentence type and age in months was also significant: with increasing age, participants were more likely to point to the causal event for transitive sentences (see Figure 3a), and less likely to point to the causal event for intransitive sentences (see Figure 3b). No other interactions were significant.

Given the significant interaction between language group and sentence type, we analysed bilingual and monolingual participants’ responses separately with sentence type and age and their interaction in months as fixed effects and sentence type as a random effect. For bilingual children, there was a significant main effect of sentence type, suggesting they were more likely to point to the causal event for transitive than intransitive sentences (Table 3, model 2).

There was no significant main effect of age in months but there was an interaction between sentence type and age in months, as older bilinguals were more likely to associate the causal event with the transitive, and less likely to associate the causal event with the intransitive, compared to younger bilinguals.

For monolingual participants, there was also a significant main effect of sentence type, as monolingual participants pointed to the causal event more often for transitive than intransitive sentences (Table 3, model 3). Like the bilingual group, there was no main effect of age but there was an interaction between sentence type and age in months, older monolinguals pointed more to the causal event for transitive trials, but less for intransitive, than younger monolinguals.

#### 3.1.1 Age Groups Analysis

**FIGURE 4:**
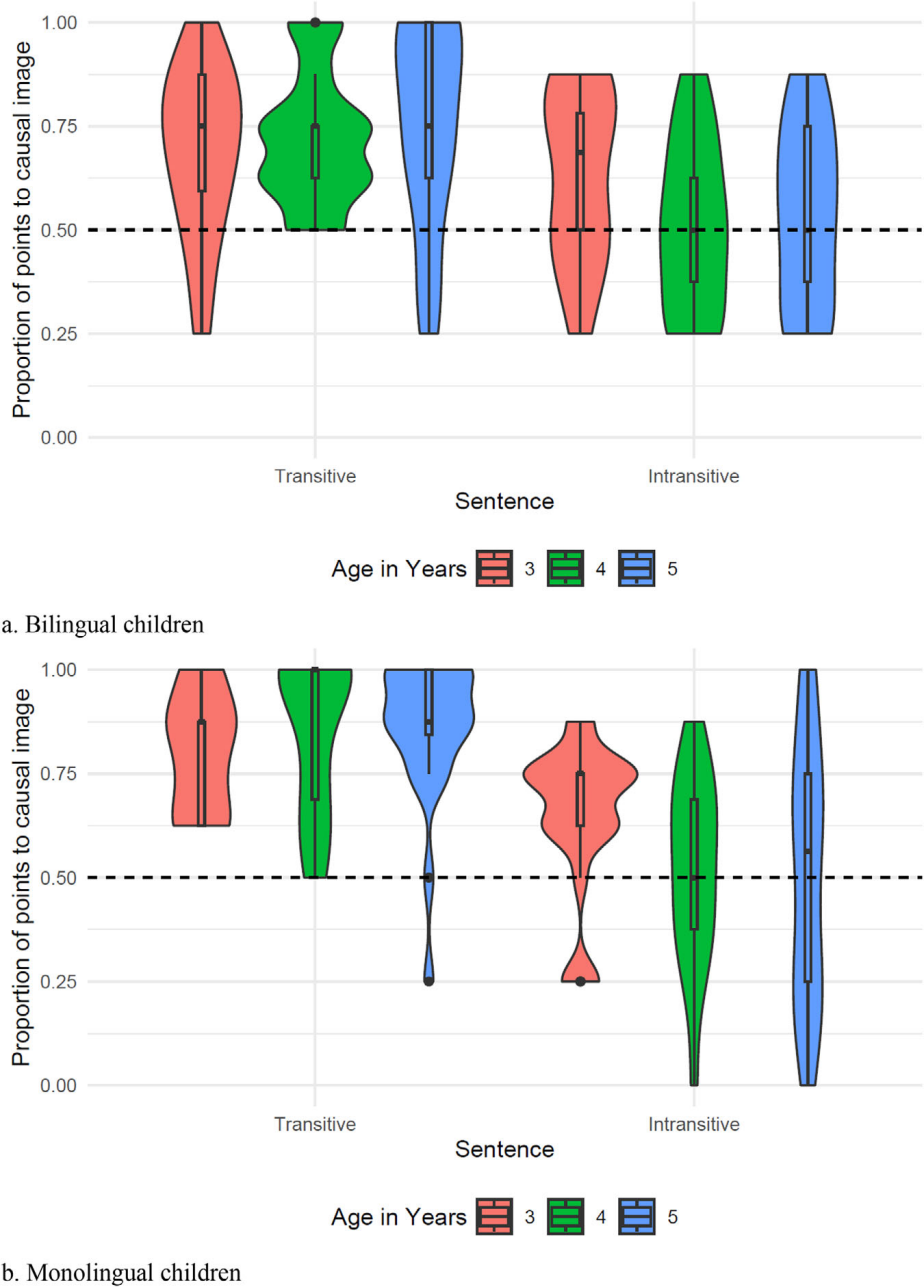
Proportion of correct points to the causal event in the sentence task for bilingual (a) and monolingual (b) participants by age. The box represents the interquartile range, the thick line represents the median, and whiskers represent the minimum and maximum scores.

To examine whether participants’ ability to point to the causal event was above or below chance, that is, whether they were interpreting transitive sentences as causal and intransitive sentences as non‐causal, we conducted a series of one‐sample *t*‐tests for 3, 4, and 5‐year‐olds in each language group, comparing the proportion of points to the causal event to chance (test statistic: 0.5). For bilingual children, we found that 3‐year‐olds’ performance was above chance for transitive sentences (*t*(15) = 3.67, *p* = 0.002), however, their points to the causal event were also above chance for intransitive sentences, (*t*(15) = 2.57, *p* = 0.021). Four‐year‐old bilinguals were above chance in interpreting transitive sentences, (*t*(12) = 4.90, *p* < 0.001), whereas for intransitive sentences, their performance did not differ from chance, (*t*(12) = 0.17, *p* = 0.870). Five‐year‐olds were also above chance for transitive sentences, (*t*(16) = 4.28, *p* < 0.001), and at chance for intransitive sentences (*t*(16) = 0.69, *p* = 0.501; see Figure 4a).

For monolingual children we found the same pattern of results: 3‐year‐olds’ performance was above chance for transitive sentences (*t*(20) = 9.93, *p* < 0.001) and intransitive sentences, (*t*(20) = 3.51, *p* = 0.002). The older children were also above chance in interpreting transitive sentences (4‐year‐olds: *t*(18) = 8.04, *p* < 0.001; 5‐year‐olds: *t*(23) = 9.89, *p* < 0.001), whereas for intransitive sentences, their performance did not differ from chance (4‐year‐olds: *t*(18) = 0.38, *p* = 0.702; 5‐year‐olds *t*(23) = 3.67, *p* = 0.807; see Figure 4b).

These analyses reveal that in both language groups, 3‐year‐olds were biased towards choosing the causal event for both sentence structures, therefore interpreting transitive sentences correctly, but conjoined agent intransitive sentences incorrectly. Four‐ and five‐year‐olds correctly associated the causal event with transitive sentences but were also able to disassociate conjoined agent intransitive sentences from a causal event, though they did not reliably associate them with the noncausal event either.

### 3.2 Relationship Between Sentence Comprehension, Vocabulary, and Executive Function

Table 4 displays the mean scores on the CLT and BST. Independent samples *t*‐tests (one‐tailed) showed a difference between bilingual and monolingual performance for the CLT, *t*(108) = −1.734, *p* = 0.043, but not for the BST, *t*(108) = −0.512, *p* = 0.305. One‐sample *t*‐tests revealed that performance on the BST was above chance levels (test statistic i.e., 15 out of 30 trials) for both bilingual, (*t*(45) = 17.74, *p* < 0.001), and monolingual, (*t*(63) = 22.81, *p* < 0.001), groups. Performance on each task increased significantly with age, with positive correlations between age in months and raw scores for CLT (*r* = 0.518, *p* < 0.001), and BST (*r* = 0.376, *p* < 0.001).

**TABLE 4:** Mean (SD) CLT verb comprehension scores (32 trials) and BST scores (30 trials) for bilingual and monolingual children across ages.

Participants	CLT score	BST score
Bilingual (all)	26.33 (4.03)	26.15 (4.26)
3‐year‐olds	24.25 (4.09)	23.81 (5.26)
4‐year‐olds	26.00 (3.81)	27.85 (1.86)
5‐year‐olds	28.53 (3.11)	27.06 (3.73)
Monolingual (all)	27.62 (3.76)	26.56 (4.06)
3‐year‐olds	24.62 (3.53)	23.86 (4.62)
4‐year‐olds	28.37 (3.10)	27.58 (2.52)
5‐year‐olds	29.67 (2.70)	28.12 (3.38)

CLT and BST were also found to positively correlate (*r* = 0.427, *p* < 0.001), therefore, we explored interactions between children's sentence processing, vocabulary, and executive function together to examine whether either factor explained the observed changes in sentence comprehension performance across age. We added CLT verb comprehension score and BST score, with both variables converted to Z scores, as fixed effects to a model with sentence type and language group (coded as in the previous analyses; Table 5). As in the model with age, there were main effects of sentence type and language group and a significant interaction between the two. There was no main effect of CLT verb comprehension score or BST score, however there was a significant interaction between sentence type and CLT verb comprehension score, suggesting that those with a higher vocabulary more frequently pointed to the causal event for transitive than for intransitive sentences. No other interactions with CLT or BST were significant. This model did not, however, provide a better fit than the model with age (*χ* ^2^ (df = 8) = 6.33, *p* = 0.610).

**TABLE 5:** Logistic mixed effects models for bilingual and monolingual sentence comprehension with vocabulary and executive function scores.^a.^ Response∼Sentence type*Language group*CLT Z Score*BST Z Score + (1+Trial|Ppt_ID) + (1|ItemNo).

Predictor	*β*	SE	95% CI	*Z*	*p*
**Model 4 – Sentence comprehension × vocabulary × executive function^a^**					
Intercept	0.84	0.12	[0.60, 1.08]	6.86	< 0.001
Sentence type	1.28	0.25	[0.79, 1.77]	5.10	< 0.001
Language group	0.32	0.16	[0.0035, 0.64]	1.98	0.047
CLT_Z score	0.10	0.08	[−0.06, 0.26]	1.19	0.233
BST_Z score	0.03	0.09	[−0.14, 0.21]	0.40	0.688
Sentence type × language group	0.81	0.34	[0.14, 1.49]	2.37	0.017
Sentence type × CLT_Z Score	0.40	0.18	[0.05, 0.75]	2.23	0.026
Language group × CLT_Z Score	−0.08	0.17	[−0.41, 0.25]	−0.46	0.642
Sentence type × BST_Z Score	−0.19	0.19	[−0.56, 0.17]	−1.05	0.294
Language group × BST_Z Score	0.13	0.18	[−0.21, 0.48]	0.76	0.444
CLT_Z Score × BST_Z Score	0.14	0.07	[−0.00161, 0.29]	1.94	0.053
Sentence type × language group × CLT_Z Score	0.34	0.36	[−0.36, 1.04]	0.96	0.336
Sentence type × language group × BST_Z Score	0.21	0.37	[−0.52, 0.94]	0.56	0.572
Sentence type × CLT_Z Score × BST_Z Score	−0.19	0.16	[−0.50, 0.11]	−1.22	0.221
Language group × CLT_Z Scores × BST_Z Scores	0.08	0.15	[−0.21, 0.37]	0.55	0.585
Sentence type × language group × CLT_Z Score × BST_Z Score	−0.27	0.31	[−0.88, 0.34]	−0.86	0.391

## 4 Discussion

This study examined bilingual and monolingual children's interpretation of transitive and intransitive sentences in English across the preschool years. We also explored whether vocabulary and executive function measures would be related to their comprehension of these sentence structures. We found few differences between the two groups: both associated causal events with transitive sentences, but monolingual children were more likely to make this association than bilingual children. There were however age‐related differences in children's comprehension of conjoined‐agent intransitive sentences: younger participants, both bilingual and monolingual, associated these sentences with causal events, whereas older participants did not distinguish between causal and non‐causal events for intransitives. We explored whether these age‐related differences were explained by executive function abilities, as measured by a non‐verbal Stroop task, the BST, or by language skills, as measured by a receptive test of verb knowledge. We did not observe any difference in executive function scores between the bilingual and monolingual groups nor any relationship between executive function and sentence comprehension performance. However, monolingual children had higher English verb vocabulary scores, and we also found that across both groups, children were more accurate on the comprehension task with increasing vocabulary scores. Our paradigm extended previous research in important ways, examining knowledge of both transitive and intransitive sentences within‐participants and across multiple (eight) trials per sentence structure. We found that young children were able to complete up to 23 trials (including pretest trials) permitting a robust measure of children's syntactic awareness.

This study presents, to the best of our knowledge, the first comparison of monolingual and bilingual children's sentence comprehension in a novel verb task. We found that 3–5‐year‐old children growing up in monolingual and bilingual environments showed similar patterns of responses when choosing the referent of a novel verb transitive and intransitive sentence: all children chose a causal event to match transitive sentences more often than would be expected by chance, which suggests little difference in bilingual children's acquisition of verb general knowledge compared to monolingual children's. However, the significant interaction between the two groups suggest that monolingual children's knowledge may be more secure at the ages tested. This is supported by the violin plots for each age group and language background which show that while monolingual children's mean points are clustered around the median suggesting less variation in their performance—most children are responding correctly—the bilingual children show a greater range of pointing behaviours, suggesting more variation in correct points and some children responding incorrectly. Another possibility is that some bilingual participants experienced crosslinguistic influence, since we did not control what languages participants spoke other than English. However, the significant interaction with vocabulary irrespective of language group indicates that performance on this task was also variable in line with children's (receptive verb) vocabulary. This relationship was observed across the two groups, but we did observe that the bilingual group had significantly lower English vocabulary scores than the monolingual group, which may also explain the differences in performance on the sentence comprehension task between groups.

This finding is consistent with other research findings showing positive correlations between language processing and vocabulary in bilingual (Hurtado et al. 2014) and monolingual children (Borovsky et al. 2012; Fernald et al. 2006; Messenger and Fisher 2018). More broadly, it also aligns with evidence that vocabulary and grammatical growth are closely linked in bilingual development (Hoff et al. 2018; Marchman and Martínez‐Sussmann 2004). These studies suggest that vocabulary knowledge may not only reflect overall language proficiency but may also support the development of grammatical representations and sentence comprehension abilities. Indeed, there may be a particularly direct link between our measure of vocabulary, which tested children's receptive verb knowledge, and the sentence comprehension task, which tested children's knowledge of verb structures. Children who have acquired more verbs in their vocabulary may also have more entrenched and abstract knowledge of the structures in which different verbs occur. Indeed, consistent with the syntactic bootstrapping hypothesis, children who have acquired abstract knowledge of structures such as the transitive may be able to use this knowledge to expand their verb vocabulary. Nonetheless, since this study did not find any substantive differences between young bilingual and monolingual children in their ability to associate causal and non‐causal events with transitive and intransitive sentences, it provides further reassurance to parents who often perceive bilingualism to be related to language delay in young children (Abutbul‐Oz and Armon‐Lotem 2022; King and Fogle 2006; Lee et al. 2015). A direction for future research would be to test whether verb‐general knowledge *first* emerges in bilingual children at the same age as monolingual children.

We found that participants, both bilingual and monolingual, did not show a preference for non‐causal events when listening to intransitive sentences. Older participants’ points were at chance which is consistent with explanations whereby a conjoined agent intransitive sentence could appropriately describe a specific action carried out by two actors simultaneously, as depicted in the non‐causal event (*the bear and the frog are bending*), or it could describe the causal event, analogous with a more general verb, (*the bear and the frog are playing*/*exercising*). These results are consistent with previous research with child participants (Arunachalam et al. 2016; Arunachalam and Waxman 2010; Noble et al. 2016), and even with adults (Arunachalam et al. 2016). Indeed, a meta‐analysis by Cao and Lewis (2022) found that across ages and amongst varying vocabulary sizes, the syntactic bootstrapping effect is reliable for transitive, but not intransitive, sentences.

Unusually, 3‐year‐old children were more likely to select the causal event on intransitive trials as well as transitive. These findings are inconsistent with previous research showing that children aged 2–3 years tend to be at or below chance for associating conjoined agent intransitive sentences with causal events (Gertner and Fisher 2012; Noble et al. 2011). It is possible that still images made it more difficult for 3‐year‐olds to ascertain that two agents were carrying out the same action in the non‐causal events, though they had no issue interpreting causal events. Alternatively, children may have misinterpreted the conjoined agent of these sentences, using the first argument as agent cue to assign the agent role to only the first noun in intransitives (Noble et al. 2016) and misinterpreting the second noun as a patient (Gertner and Fisher 2012). These errors may reflect developing knowledge of conjoined agents. Crucially, monolingual and bilingual children showed the same pattern of behaviour suggesting that bilingual children's development of verb‐general knowledge is not qualitatively different from that of monolingual children's.

Indeed, the overall patterns of performance were very similar between the two groups in this study, including on the executive function tests, and though monolingual children's English vocabularies were significantly higher, the mean difference between groups was small. One possible explanation is that the children in this study mostly came from high‐SES backgrounds. Research has found that high‐SES children typically outperform low‐SES children on vocabulary measures (Bialystok et al. 2010; Calvo and Bialystok 2014; Hoff 2003). The lack of relationship with executive function skills may also be due to the high‐SES backgrounds of participants; Calvo and Bialystok (2014) found that high‐SES children outperformed low‐SES children on tests of executive function while Huang et al. (2017) found SES‐related differences in monolingual children's sentence processing. Thus, our results may be specific to children from higher‐SES backgrounds. Future research could build on these findings by systematically investigating SES; in the UK, many bilingual children come from low‐SES households, since many bilingual households are also immigrant and ethnic‐minority families that may have limited English language skills or lower household income (Matejic et al. 2024; Qureshi and Morris 2025).

Another direction for future research would be to test a larger sample of children. It is possible that the null result for executive function could be due to low statistical power to detect an effect, though we note that, when examining performance without age, we were able to detect an effect of vocabulary as a significant predictor of points to the transitive events on transitive, versus, intransitive trials. Nonetheless, a limitation of this study is that our sample size was based on those of previous research and we did not quite reach the desired total for the bilingual sample, though unlike these studies, we tested sentence structure as a within‐participants not between‐participants manipulation. Moreover, most previous studies primarily examined performance on the sentence comprehension task rather than how this relates to other factors. While this sample size may be sufficient to detect effects on this one task, it is possible that to observe robust relationships between individual differences measures, such as vocabulary and executive function, and performance on the sentence comprehension task, a larger sample with greater variability and greater power to detect relationships is required. Our overall sample was relatively large (46 bilingual children and 57 monolingual children), however, this was spread across three years of age such that the contribution of each factor over and above age could be difficult to observe (indeed we found that the model with executive function and vocabulary measures was not a better fit of the data than the model with age). Collecting larger samples within each age band would permit a more robust observation of the relationship between sentence comprehension and individual differences other than age.

As this study was conducted online, a potential limitation could be the lack of a controlled environment during testing: despite repeated reminders during the tasks that parents should not help their child to point to the correct answers, we cannot be sure that this was the case, nor can we be sure that testing took place in a quiet environment. However, other developmental research has shown comparable findings in online data collection to those from in‐person studies (Buckle et al. 2025; Scott et al. 2017). Since we did not observe any responses that were 100% correct across all tasks for any participant, we assume that these results can be taken on trust.

Moreover, conducting research online did allow for a varied bilingual sample to be reached. Though the focus of this study was on English, the study included bilingual children from a diverse range of spoken languages, with 18 languages represented among the bilingual participants. These participants were representative of the bilingual population in the UK, where most of our participants came from, as all of the ten languages (other than English or Welsh) most‐spoken in England and Wales were included in this study (Waddington 2022). While we did not control for language background in order to reach a wider sample more easily, future research could target bilingual children from specific language backgrounds to investigate whether and how crosslinguistic influence may play a role in the development of early verb‐general knowledge of syntactic structures. Future research could also collect more data from bilingual families on the amount of exposure to each of their languages. Since this study was conducted remotely, we did not wish to further increase the time taken to participate, in order to ensure parental and child engagement and completion of the study. However, such information may be useful for understanding variation in performance within and across groups.

## 5 Conclusion

In this study, we showed that bilingual and monolingual preschoolers could flexibly switch between novel verb transitive and intransitive sentences across a large number of experimental trials. In addition to demonstrating that young children can complete more extensive experiments, which should provide more reliable measures of their early comprehension abilities, we conducted the first such test of early grammatical knowledge in bilingual as well as monolingual learners. We found clear evidence that, irrespective of language background, preschoolers have verb‐general knowledge for transitive sentences, though their ability to demonstrate this, increases with age and vocabulary growth. Monolingual children had higher English vocabulary scores and were more reliably accurate on the sentence comprehension task than bilingual children. Despite this, both groups showed above chance performance at the same ages. Furthermore, in both groups, older children's pointing patterns for intransitive sentences were consistent with past research which suggests that either causal or non‐causal scenes can provide a plausible referent for such sentences, whereas younger children's pointing patterns suggested systematic errors in interpreting these sentences, likely due to the conjoined agent (Gertner and Fisher 2012). Our results suggest that further research into the relationship between SES and sentence comprehension is warranted; this may be particularly pertinent in the context of bilingual language development. Future work should also examine development of sentence comprehension within the heritage language, since here we focused solely on the societal language (English). Nonetheless, consistent with recent research showing bilingual and monolingual children reach a variety of milestones at the same ages (Höhle et al. 2020; Muszyńska et al. 2026), our results provide positive evidence for similar comprehension performance across bilingual and monolingual preschoolers, which should allay concerns of particular language delay in bilingual development.

## Author Contributions

**Katherine Messenger**: supervision, conceptualization, investigation, funding acquisition, writing – original draft, methodology, validation, writing – review and editing, formal analysis, software, project administration, data curation, resources. **Noorin Rodenhurst**: conceptualization, investigation, funding acquisition, writing – original draft, methodology, validation, visualization, writing – review and editing, software, formal analysis, project administration, data curation, resources.

## Funding

This research was funded by a University of Warwick departmental studentship to the first author.

## Ethics Statement

This research was reviewed and given approval by the University of Warwick Research Ethics Committee.

## Conflicts of Interest

The authors declare no conflicts of interest.

## Permission to Reproduce Material

Permission to reproduce material from other sources: Permission to reproduce material from Noble and Rowland (2011) in Figure 1 was provided by Claire Noble and Caroline Rowland.

## Data Availability

The raw data, analysis code and model outputs are available on OSF https://osf.io/gdvcu/?view_only=82d1e2aad30c488ea767303da6597e4f

## Endnotes

1 Twenty‐four participants completed a first version of the task without any character ID trials – to avoid participant fatigue due to the increased number of trials, the four character ID trials at the start of the original task were initially removed, however, initial data showed that some participants (6/24) were not passing the screening trials, therefore we re‐inserted two trials at the start of the task. 94 participants completed this version.

## Appendix

The following Table A1 contains all the pretest and test sentences used during the sentence comprehension task, in the order they were displayed to participants.

**TABLE A1:** Order of trials and items with target response and sentence stimuli.

Trial	Pre‐test item	Correct response	Sentences
Character ID 1	Frog	Right	Where's the frog? Point to the frog.
Character ID 2	Rabbit	Left	Where's the rabbit? Point to the rabbit.
Real verb 1	Feed	Left	Look, the bear is feeding the frog. Point to where the bear is feeding the frog.
Real verb 2	Wash	Right	Look, the frog is washing the bear. Point to where the frog is washing the bear.
Screening 1	Norp	Left	Look, the bear is norping. Point to where the bear is norping
Screening 2	Rax	Right	Look, the sheep is raxing. Point to where the sheep is raxing.
Screening 3	Grad	Left	Look, the duck is gradding. Point to where the duck is gradding.

Trial	Test item	Transitive image	Sentences
Intransitive 1	Ginde	Right	Look, the duck and the rabbit are ginding. Point to where the duck and the rabbit are ginding.
Intransitive 2	Blick	Left	Look, the duck and the rabbit are blicking. Point to where the duck and the rabbit are blicking.
Transitive 1	Glorp	Right	Look, the rabbit is glorping the duck. Point to where the rabbit is glorping the duck.
Transitive 2	Meek	Right	Look, the rabbit is meeking the duck. Point to where the rabbit is meeking the duck.
Intransitive 3	Nav	Right	Look, the duck and the rabbit are navving. Point to where the duck and the rabbit are navving.
Intransitive 4	Pake	Left	Look, the duck and the rabbit are paking. Point to where the duck and the rabbit are paking.
Transitive 3	Wug	Right	Look, the rabbit is wugging the duck. Point to where the rabbit is wugging the duck.
Transitive 4	Klimp	Left	Look, the duck is klimping the rabbit. Point to where the duck is klimping the rabbit.
Intransitive 5	Filp	Right	Look, the duck and the rabbit are filping. Point to where the duck and the rabbit are filping.
Intransitive 6	Pilk	Left	Look, the duck and the rabbit are pilking. Point to where the duck and the rabbit are pilking.
Transitive 5	Rill	Right	Look, the duck is rilling the rabbit. Point to where the duck is rilling the rabbit.
Transitive 6	Moop	Left	Look, the rabbit is mooping the duck. Point to where the rabbit is mooping the duck.
Intransitive 7	Dax	Left	Look, the duck and the rabbit are daxing. Point to where the duck and the rabbit are daxing.
Intransitive 8	Krad	Right	Look, the duck and the rabbit are kradding. Point to where the duck and the rabbit are kradding.
Transitive 7	Jemm	Left	Look, the duck is jemming the rabbit. Point to where the duck is jemming the rabbit.
Transitive 8	Jeed	Left	Look, the rabbit is jeeding the duck. Point to where the rabbit is jeeding the duck.
